# Systematic assessment for potential endogenous normalizers for microRNA analysis in fecal samples

**DOI:** 10.1038/s41598-026-57583-2

**Published:** 2026-06-25

**Authors:** Theresa Lederer, Konrad Lehr, Cosima Thon, Katharina Magin, Andreas Jeron, Denny Schanze, Martin Zenker, Ali Canbay, Alexander Link

**Affiliations:** 1 Department of Gastroenterology, Hepatology and Infectious Diseases, Otto-von-Guericke University, Leipziger Str. 44, 39104 Magdeburg, Germany; 2 Molecular Gastroenterology and Microbiome Research Unit, Institute for University Teaching and Research Medical Campus Upper Franconia, Friedrich-Alexander University Erlangen-Nürnberg, Bayreuth, Germany; 3 Institute of Medical Microbiology and Hospital Hygiene, Infection Immunology Group, Health Campus Immunology, Infectiology and Inflammation, Otto-von-Guericke University, Magdeburg, Germany; 4 Institute of Human Genetics, Otto-von-Guericke University, Magdeburg, Germany; 5 Medizinische Klinik, Universitätsklinikum der Ruhr-Universität Bochum, Bochum, Germany; 6 Department of Gastroenterology, Friedrich-Alexander University Erlangen-Nürnberg, Medical Campus Upper Franconia, Klinikum Bayreuth, Preuschwitzer Str. 101, 95445 Bayreuth, Germany

**Keywords:** Biomarkers, Cancer, Computational biology and bioinformatics, Diseases, Gastroenterology

## Abstract

MicroRNAs (miRNA) are considered promising biomarkers for inflammatory and neoplastic diseases in fecal samples, but despite reproducible miRNA detection, there is no consensus on appropriate normalization in fecal samples. In this study, we aimed to explore an endogenous normalizer for miRNA analysis in fecal samples. For this purpose, we performed a multistep study with 3 independent cohorts. In the first step, we investigated miRNA profiling using serial dilutions of blood and fecal samples from healthy subjects. In the second part, we tested the analysis using a cohort of IBD patients (*n* = 30) with active disease and in remission and 59 patients with different liver pathologies or healthy subjects. Finally, the data were validated using an independent cohort of patients with liver disease (*n* = 360). After extraction, miRNAs were analyzed using Affymetrix GeneChip microarray technology in the screening and confirmation cohorts, and TaqMan qPCR was performed for validation. Analysis of the blood stool mixture cohort revealed 5 miRNAs that were stable in all three subgroups. Among them, miR-638 was found to have a Log_2_FC of 0.0004 and was selected for further analysis as one of the best studied miRNAs from previous reports. The stability of miR-638 in microarray analysis was demonstrated in two independent cohorts of patients with IBD and liver disease. We observed no effect of age or other factors on miR-638 levels in fecal samples. Furthermore, its relatively high concentration in feces and its stability against potential blood contamination may offer great advantages over the previously used miR-16, suggesting miR-638 as a potential endogenous fecal normalizer.

**Supplementary Information:**

The online version contains supplementary material available at 10.1038/s41598-026-57583-2.

## Introduction

MicroRNAs (miRNA) are small non-coding RNAs that are involved in post-transcriptional regulation and protein translation and play a critical role in the development and prevention of numerous diseases.^1–4^ They were first described in various aspects of leukemia^5^, but development has been rapid, and it is well established that miRNAs play an important role in almost every biological process in the body and could be used even for therapeutic strategies in various diseases. ^5–8^

Most importantly, miRNA have specific properties that suggest them as potential biomarkers in multiple human diseases. However, in the transition to clinical implementation, it is therefore crucial to optimize the analytical methods for detection of miRNA for diagnostic and therapeutic purposes. There are numerous ways to study the concentration and function of miRNAs in different body fluids and tissues. The most commonly used method is extraction followed by real-time PCR, complemented by microarray analysis or sequencing.^9^ One of the problems related to qPCR-based analysis is the need of a stable endogenous normalizer with equal concentrations in different body fluids and tissues, since the concentration of miRNAs in blood or tissue can vary tremendously.^2,10^ While, the normalization of miRNA in tissues is commonly performed using RNU6b, for the blood or fecal specimens, there are currently no standardized methods, while it is clear that RNU6b has substantial differences in biogenesis and may not be used as a standard^2^. Currently, the non-human miRNA cel-miR-39 from *Caenorhabditis elegans* is commonly used as an artificial spiked-in normalizer during extraction that can be precisely measured during subsequent steps.^10,11^ The use of endogenous normalizers is especially interesting in diseases that have a high risk of contamination of specimens that are not reflecting the local biogenesis, for instance feces with blood, like colorectal cancer (CRC) or Inflammatory bowel diseases (IBD)^1,12–14^.

Probably the longest history in fecal miRNA research is related to the colorectal cancer (CRC)^1,15^. According to the pioneer work in this field, there is a potential risk of bias due to blood contamination of the fecal sample from microbleeds, which are common in diseases with colon pathology^1,15^. For instance, an effort has been made to use RNU6b, but it is prone to higher levels of degradation and has different biology and therefore miR-16 and miR-26b have been utilized as alternatives. Nevertheless, these two may also be inadequate when the samples are contaminated with blood, which could result in biased outcomes.^1^ At present there is no consensus on miRNAs for normalization.^10^ Therefore, it is of substantial advantage to identify an endogenous marker for normalization of fecal miRNAs that is not affected by blood contamination and that could contribute to the reliability of the data^10^.

This issue has been further investigated in IBD, which is a chronic inflammatory disease with a substantial need for potential biomarkers^16–18^. It is well known that one of the causes of symptoms in IBD patients is mucosal leakage as well as microbleeds due to the destruction of epithelial structures through a variety of immune cells and factors^13,14^. IBD is also commonly associated with minor or even major events of bleeding due to severe colitis and therefore may be considered as biased for blood contamination^13,14^. Therefore, it is essential to adapt the biomarker to these conditions and to capture the preanalytical factors. As a result, the use of spiked-in cel-miR-39 has also been adapted for the fecal specimen, but due to potential dilution in watery diarrhea it may also not be the perfect normalizer due to its synthetic origin and inability to reflect the steady state of the fecal miRNA biogenesis in the gut^10^.

Furthermore, it is important to note that research on miRNAs in fecal samples has expanded beyond their role in gut-related diseases. Nowadays, organs and diseases with no direct connection to the gut, and therefore feces, are also being studied.^4,19^ Liver pathologies are also receiving increased attention, but there is a lack of data regarding miRNA in fecal samples.^20–24^ Metabolic dysfunction associated steatotic liver disease (MASLD) and alcohol associated liver disease (ALD) are the leading causes of liver-related mortality in modern countries.^25–27^ Therefore, further development of early-stage diagnostics and therapies is crucial. Small non-coding RNAs, such as miRNAs, play an important role in the development and prevention of these liver diseases and could be used as potential biomarkers or therapeutic targets.^20,28^.

In this study, we aimed to identify a potential endogenous miRNA normalizer for the analysis of miRNA in feces. For this purpose, we performed a systematic analysis using a three-step approach which included implementation of a unique artificial model to imitate the contamination using serial dilution of blood in feces to exclude potential blood-related impact and included two steps with testing and validation using two independent cohorts of patients with IBD and liver diseases.

## Results

### Impact of blood contamination on the miRNA quantity

**Fig. 1:**
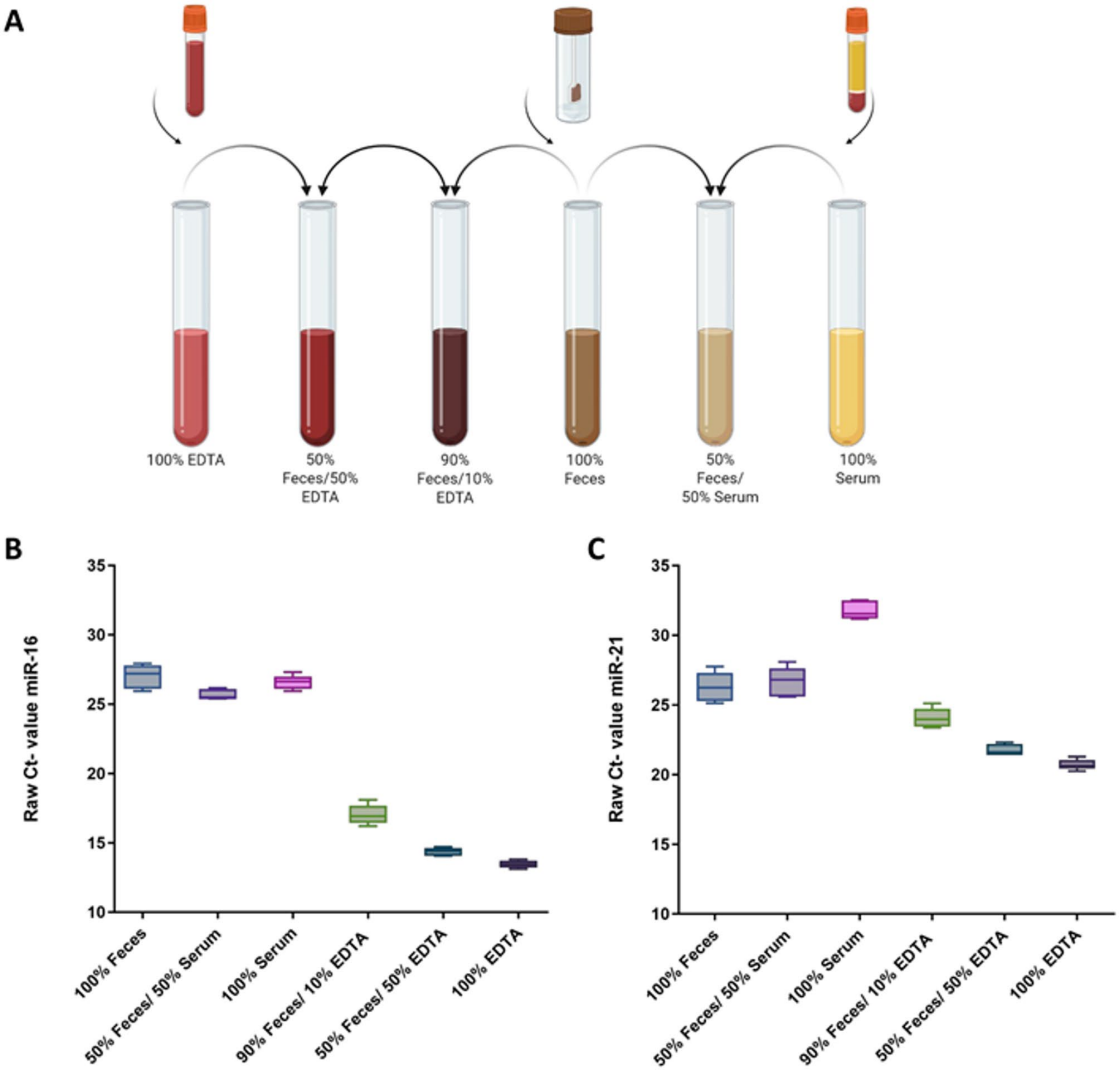
Serial dilution of blood and feces. **(A)** Workflow. *Created in BioRender. Link*,* A. (2026)* https://BioRender.com/soyx2fg**(B)** Serial dilution of miR-16. **(C)** Serial dilution of miR-21.

With the knowledge that various diseases such as CRC or IBD may be associated with obscure or overt amount of blood in feces, there is a great need for a study model that would help to improve the assessment of miRNA levels without potential bias of those alterations. With this aim, to enhance our understanding of the occult or overt bleeding on the fecal samples, we conducted serial dilution experiments using systematically processed fecal specimens and for the proof-of-principle purpose we analyzed most studied miRNA such as miR-21 and miR-16 for its high levels in erythrocytes. Fecal samples were diluted using various ratios, including 100% fecal sample, 50% feces and 50% serum, 100% serum, 90% feces and 10% EDTA blood, 50% EDTA blood and 50% feces, and 100% EDTA blood (Fig. 1A). Here we observed that the highest concentration of miR-16 and miR-21 were found in pure blood (EDTA), while serial dilution resulted in systematic decrease of miR-21 and miR-16 for the mix with 50% and 10% EDTA blood mixed with feces (Fig. 1B**/C**). Interestingly, the level of studied miRNA was intermediate in pure fecal specimens and following addition of serum samples (artificially mimicking the leaky barrier) the level further decreased. Overall, the lowest level of miR-21 was in serum samples while miR-16 was somewhat comparable between fecal and serum specimens. Thus, the data clearly support the impact of occult or overt bleeding events, such as the case in CRC or IBD with a shift in miRNA alteration particular of miR-16 that has been used for normalization in the past.

### Unbiased screening for potential endogenous normalizing miRNA

**Fig. 2:**
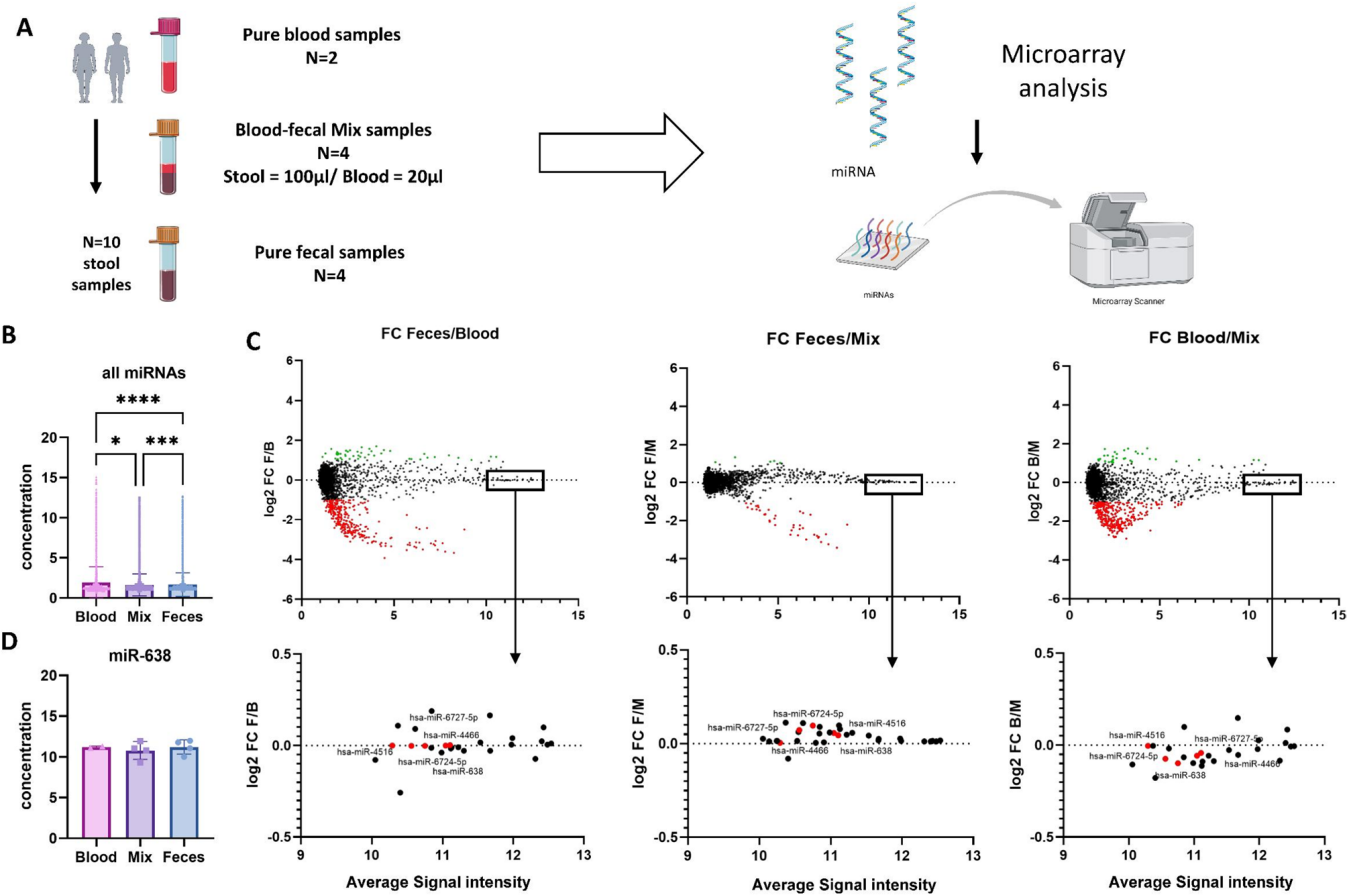
Screening cohort. **(A)** Workflow. Created in BioRender. Link, A. (2026) https://BioRender.com/xloh8ab**(B)** Comparison of the concentration of miRNAs in the different groups. Kruskal-Wallis Test, *p = 0.05, **p = 0.01, ***p = 0.001, ****p = 0.0001. **(C)** Log_2_FC analysis of the groups. with focus on the highest signal intensities and least different miRNAs. Top: red: Log_2_<-1 green: Log_2_>1, Bottom: in red mentioned in text **(D)** Concentration of miR-638 in all groups.

Having shown the substantial impact of blood contamination on the miRNA analyses in feces, we next performed the unbiased microarray screening in a cohort of pure blood, blood and fecal mix samples and pure fecal samples from healthy patients to identify the potential miRNAs that may remain stable independent of the blood contamination in fecal specimens using a similar approach as described above (Fig. 2A and B). As expected, highly significant differences were observed between different subgroups in relation to blood concentration (blood/feces p-< 0.0001, blood/mix *p* = 0.0155). The lowest concentration of miRNAs was found in feces-only samples (Feces/Mix p-value = 0.0003). To evaluate the concentration pattern of each miRNA, in the next step a fold change analysis was performed for the different groups (Fig. 2C). This analysis maintained the overall ratio shown in Fig. 2B. When comparing fecal versus blood samples, multiple miRNAs showed higher levels in blood, including commonly used normalizers such as miR-16, miR-26b-5p, and miR-106a (log_2_FC miR-16 = -3.67, log_2_FC miR-26b = -2.22, log_2_FC miR-106a = -3.23). At the same time, there were only few miRNAs that were detected in feces at higher abundance than in blood. The same applies to the comparison between stool and mixed samples and between blood and mixed samples. With this knowledge we further focused on the miRNA that showed greatest stability independent on influencing factors. The analysis of these miRNAs was deepened by narrowing the search and focusing on the miRNAs with a log_2_FC value less than 0.5 and a signal intensity greater than 10 (Fig. 2C). This led to identification of 5 miRNAs with low log_2_FC values such as miR-638 (log_2_FC = 0.00047), miR-4466 (log_2_FC = 0.0029), miR-4516 (log_2_FC = 0.00043), miR-6727-5p (log_2_FC = -0.0011), and miR-6724-5p (log_2_FC = 0.00097). Since these miRNAs were not affected by the mix of blood in the stool, we hypothesized that they could serve as potential internal miRNA normalizer of human origin probably primarily originating from the GI tract in comparison to frequently used miRNA with high concentration in blood. Because of the increasing appreciation of miR-638 in available literature^19,29^ and the still limited knowledge on the other miRNAs, the focus for subsequent analyses was set on miR-638 for further analysis. Similar to the previous experiments, all samples contain the same concentration of miR-638 (Fig. 2D).

### Confirmation stability of miR-638 in different patient cohorts

**Fig. 3:**
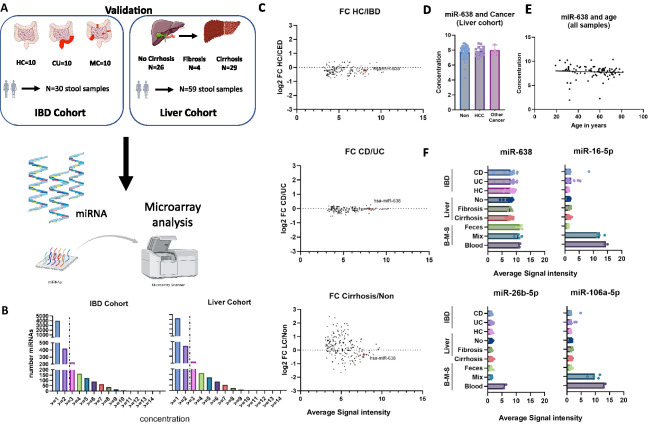
Microarray investigation. **(A)** Workflow. Created in BioRender. Link, A. (2026) https://BioRender.com/xloh8ab**(B)** General concentration and distribution of miRNAs expression in the 2 validation cohorts. Set cut-off at 3. **(C)** Log_2_FC analysis of HC against IBD, Crohn‘s Disease (CD) against Ulcerative colitis (UC) and Cirrhosis against no cirrhosis. **(D)** Comparison of signal intensity of miR-638 and cancer in the liver cohort. **(E)** Correlation of miR-638 and age with all samples, investigation cohort included. **(F)** Comparison of miR-638 with already known miRNAs.

To validate if the selected miRNA may be of potential value for the miRNA normalization, we performed subsequent miRNA array analysis in two independent cohorts with IBD patients, patients with liver pathologies and healthy subjects (IBD: *n* = 20, liver pathology: *n* = 59, healthy: *n* = 10, Fig. 3A). First, we filtered out miRNA with low and very low microarray signals out of the analysis and focused on the miRNA with log2 signal intensity > 3 in at least 50% of the cohort samples to ensure a sufficient overall miRNA concentration level. This resulted in 160 miRNAs in the IBD-patients cohort and 202 miRNAs in the patients from the liver patient cohort (Fig. 3B). Remaining miRNAs were analyzed for smallest differential concentration between cohorts. We found miR-638 to be stably concentrated in both the healthy and the IBD cohort with a marginal fold difference (log_2_FC=-0.187). Despite the expectedly higher risk for blood contamination of feces samples from the UC cohort, miR-638 was also stably concentrated with little differential variation comparing UC to Crohn´s Disease (CD) patients (CD/UC: log_2_FC= -0.021, Fig. 3C). Comparable results were observed in the liver patient cohort with or without liver cirrhosis (log_2_FC= -0.339, Fig. 3C). These initial findings indicated miR-638 to be a promising miRNA candidate for normalization purposes.

Based on the preliminary description of miR-638 indicating to have potential tumor-suppressive functions, its involvement in carcinogenesis and its negative association with age^19,29^, we performed specific subgroup analyses. No significant differences were observed between patients with or without hepatocellular carcinoma (HCC), and other subgroups in the liver patient cohort (Fig. 3D). Nevertheless, there is a slight negative correlation between age and miR-638 levels (*r*=-0.235/ *p* = 0.029, Fig. 3E).

Next, we examined the levels of other miRNAs, previously used as normalizers, in all cohorts and subgroups, including miR-16, miR-26b-5p, and miR-106a, which have been reported as reference miRNAs in prior studies.^1–4,10^ As shown in Fig. 3F, the previously described miRNAs had either low signal intensities determined by Microarray or were biased by the contamination of blood in the stool sample. Therefore, the use of miR-638 as a possible normalizer could have potential advantages over the previously proposed miRNAs that can overcome the limitations of the other miRNAs.

### Validation of miR-638 with independent RT-PCR

**Fig. 4:**
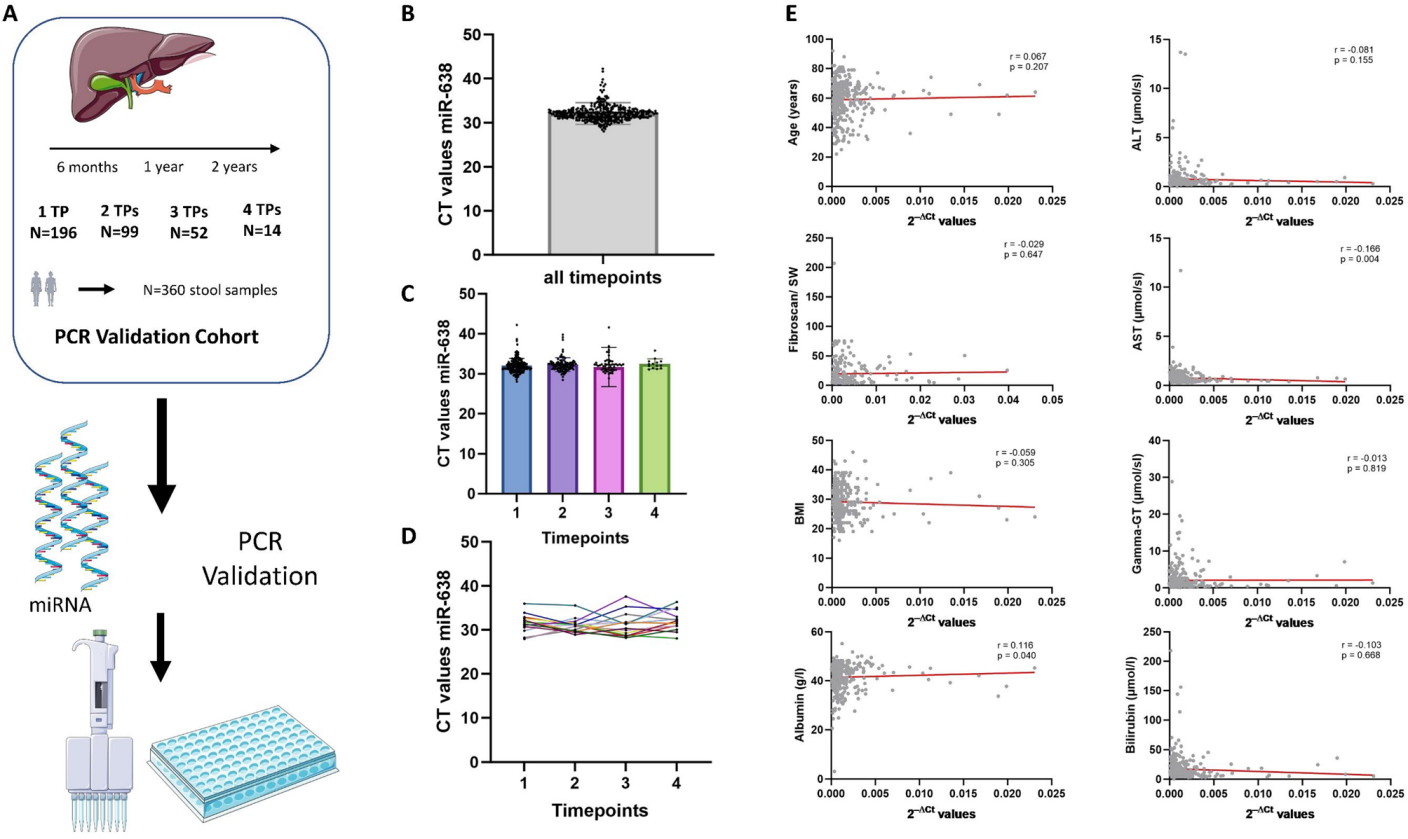
Independent RT-PCR validation. **(A)** Workflow. (**B)** Overview of the raw CT values of all PCR samples. **(C)** Distribution of raw CT values over the 4 timepoints. **(D)** 14 samples with 4 timepoints and the course of miR-638 over 2 years. **(E)** Spearman correlations with different cofactors.

Having confirmed a potential miRNA for normalization, we further aimed to validate the results also using qPCR analysis (Fig. 4A**).** For validation purposes, 360 stool samples from patients with various liver diseases were analyzed to confirm the results. The average range of CT values for the samples were between 28 and 38 (Fig. 4B). No major differences were observed when comparing different time points (Fig. 4C), and the expression level was found to be stable over time when compared across the different time points (Fig. 4C & D). No differences were observed between the liver disease groups based on etiology such as ALD and MASLD. There were also no significant differences between the different stages of liver fibrosis or cirrhosis (Supplementary Fig. 1).

Spearman correlation was used to examine the dependence of miRNA-638 on various laboratory values, liver stiffness and age (Fig. 4E). The significant negative correlation with age observed in the microarray analysis (Fig. 3D) was not confirmed in the PCR validation. Similarly, no significant correlations were found for liver stiffness, BMI, ALT, gamma-GT, and bilirubin. 2^−∆CT^ of miR-638 showed only a weak but significant negative correlation with AST (*p* = 0.004, *r*=- 0.166) and albumin (*p* = 0.040, *r* = 0.116), which demonstrates the overall independence of miR-638 from environmental factors.

### Application of miR-638 as normalizer and comparison with cel-mir-39

**Fig. 5:**
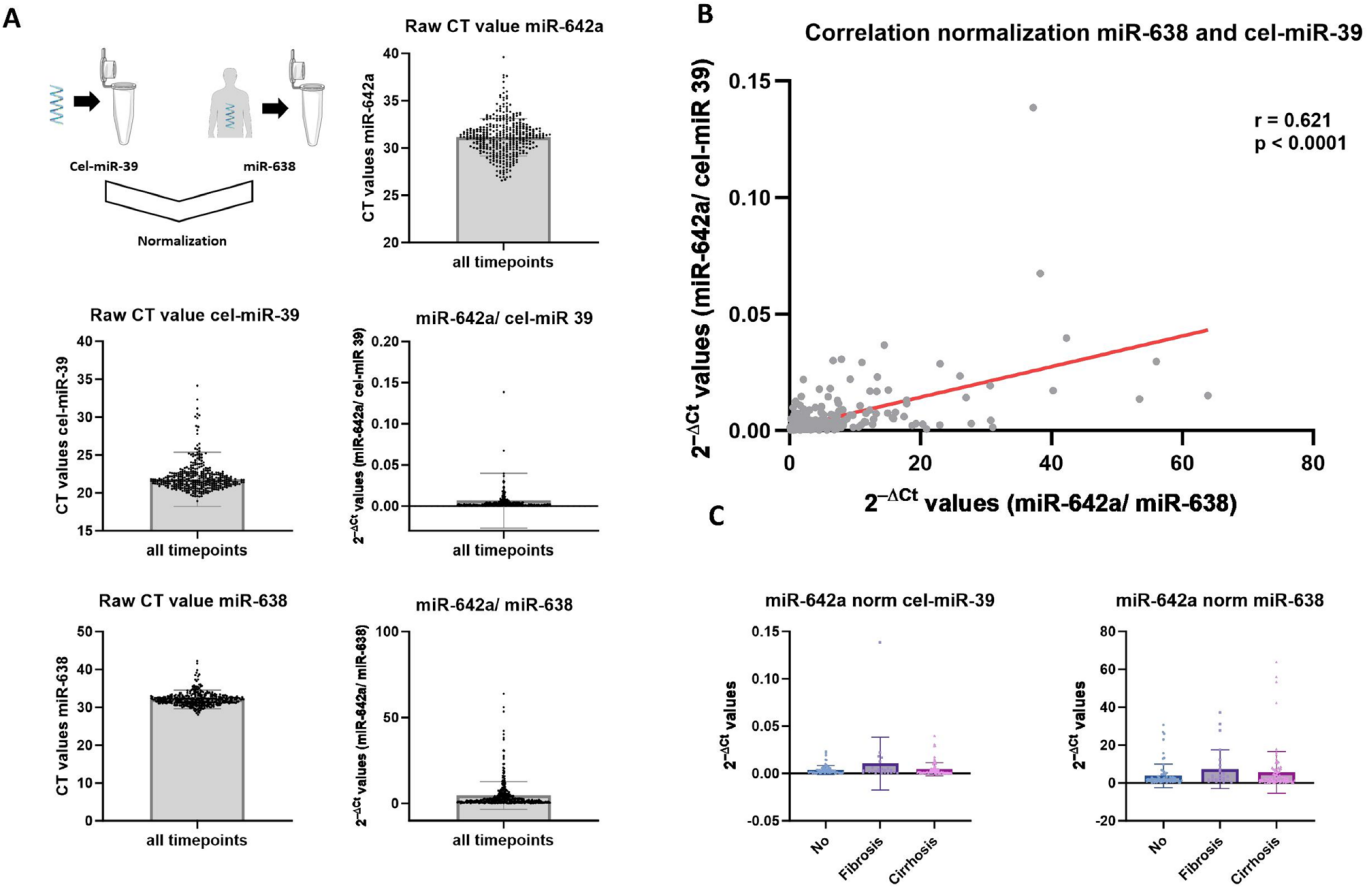
Comparison of endogenous and exogenous normalizers. (**A**) Comparison of cel-miR-39 and miR-638 and miR-642a, raw CT values and normalization comparison with cel-miR-39 and miR-38. (**B**) Spearman correlation of 2^−∆CT^ values of miR-642a normalized with cel-miR-39 and miR-638. (**C**) Comparison of the normalizers using liver disease progression.

Finally, to directly evaluate the applicability of miR-638 in a real-world normalization setting, we compared its performance with the widely used exogenous spike-in control cel-miR-39 using an independent target miRNA (miR-642a) in a clinical liver disease cohort. We performed a separate experiment with the same sample from the PCR validation cohort in Fig. 4 to demonstrate the applicability of our new normalizer to an independent miRNA. For this purpose, we selected miR-642a as an independent miRNA for testing, which has been previously described in the context of liver pathologies^30,31^ and cancers^32,33^, showing promising biomarker abilities and opening a new research field for liver pathologies in future studies. We first compared the raw CT values of all the involved miRNAs, in detail miR-642a, miR-638 and cel-miR-39 (Fig. 5A), which resulted in a significantly higher concentration of cel-miR-39 than the other two miRNAs. The 2^−∆CT^ method reveals significant differences in the 2^−∆CT^ values between miR-638 and cel-miR-39, which can be attributed to the high concentration differences in the raw CT values. To compare the normalization abilities of the two normalizers, we correlated their 2^−∆CT^ values and found a highly significant correlation (*r* = 0.621, *p* < 0.0001), indicating similar normalization behavior (Fig. 5B). To ensure applicability in a clinical setting, we compared the normalizers in an analysis of different liver disease stages (Fig. 5C). No significant differences were observed between the liver disease stages, irrespective of the normalizer employed. This finding further substantiates the comparability of both normalizers, with miR-638 however exhibiting a distinct advantage due to its inherent expression in patient material without the need for spiking-in normalizing miRNAs. Normalization using miR-638 yielded highly comparable results to cel-miR-39 across different liver disease stages, supporting its robustness and suitability as an endogenous fecal reference miRNA.

## Discussion

One of the main challenges in miRNA analysis is the choice of an optimal normalization strategy, particularly considering potential bias from blood contamination in conditions such as CRC or IBD.^1,12–14^ In this study, we demonstrated the impact of blood contamination on miRNA concentration and identified and evaluated a new normalizer for miRNA analysis.

While previously speculated, our data highlight the challenges of using miRNAs as biomarkers and emphasize the need for accurate and reproducible methods for clinical application. Notably, studies on fecal miRNAs proposed miR-16 and miR-26b-5p as potential normalizers in stool samples for CRC, although their suitability may vary across clinical contexts.^1^ This may also explain why decreased miR-21 levels were observed in CRC patients compared to those with adenomas^1^. Similar findings were reported by Faraldi et al., and a meta-analysis additionally identified miR-106a as a normalizer specifically for GI tumors.^10^ Thus, as previously suggested and now clearly validated, common markers—particularly those highly expressed in blood—may have limited value as normalizers.^10,34^

Using a unique ex vivo study design, we characterized the impact of blood contamination on fecal miRNA concentrations and identified five potential endogenous normalizers. Subsequent validation across independent cohorts and different analytical methods confirmed miR-638 as a particularly suitable candidate. An optimal normalizer should display stable concentrations across diseases and disease progression while reflecting preanalytical factors such as sample collection, quantity, and storage^10^. Unlike exogenous controls such as cel-miR-39, miR-638 fulfilled these criteria, showing both stability across liver disease groups and comparability to cel-miR-39. Importantly, the comparison with cel-miR-39 should be interpreted cautiously, since both normalization strategies were applied to the same target miRNA. Therefore, this analysis primarily illustrates the practical feasibility and consistency of miR-638-based normalization rather than demonstrating full equivalence between normalization approaches. Further studies are needed to confirm its robustness in other liver diseases and in additional clinical conditions.

In addition to etiological factors, other aspects must be considered. Francavilla et al. reported a negative correlation between miR-638 and age in healthy stool samples,^19^ whereas Hu et al. found no such association in GI cancer tissues.^29^ Our microarray analysis suggested an age-related effect, but this was not confirmed in an independent qPCR-based validation. Moreover, miR-638 has been described as a tumor suppressor in various cancers, with downregulation linked to poorer outcomes and its potential as a therapeutic target.^29,35–38^ In our fecal cohorts, however, no differences between cancer subgroups were observed, though this does not exclude functional roles in blood or tissues. Importantly, we demonstrated the long-term stability of miR-638 in fecal samples over a two-year follow-up-period. To our knowledge, previously this has not been systematically reported for stool samples, as most stability analyses have focused on blood or tissue samples.^39–41^ One possible explanation for the observed stability of miR-638 in fecal samples despite its reported involvement in tumor biology may be the compartment-specific origin and regulation of fecal miRNAs. While miR-638 dysregulation has been described in tumor tissues and circulating blood, fecal miRNA composition likely reflects a complex mixture of epithelial shedding, microbiome interaction, intestinal secretion, and dietary contributions. Therefore, disease-associated alterations observed in tissue may not necessarily translate into measurable variability in fecal specimens. Moreover, the absence of reproducible associations with age or tumor status in our independent qPCR validation cohort suggests that miR-638 variability in feces is limited compared with other biological compartments.

In recent years, understanding of global miRNA biogenesis has expanded. Notably, miRNAs have been identified in food, suggesting that ingested dietary components may influence fecal miRNA levels^42^. The analysis of exogenous xeno-miRNAs in pathophysiology further underscores the need for endogenous biomarkers unaffected by external factors. Future studies of xeno-miRNAs, such as the recently reported miR-168, may provide additional insights and contribute to more reliable and reproducible results.

Despite the systematic and detailed approach of our study, several limitations need to be acknowledged. Independent validation in larger, multicenter cohorts will be essential to confirm our findings, particularly since qPCR and microarray methods are subject to considerable variability between laboratories and may eventually be replaced or complemented by sequencing technologies. Furthermore, our analyses were restricted to a limited range of gastrointestinal diseases, and additional studies in broader patient populations are required. Expanding investigations into other specimen types—including urine, blood components, saliva, cerebrospinal fluid, and tissues from various organs—would further strengthen the understanding of the stability and biogenesis of miR-638 and other candidate miRNAs in a systematic manner.

In conclusion, we performed a comprehensive, multi-step strategy to identify potential endogenous normalizers for fecal miRNA analysis. Our results clearly demonstrate that fecal miRNA levels can be influenced by blood contamination and that the use of previously applied normalizers may introduce bias under such conditions. By combining serial dilution experiments, analyses across multiple cohorts, and independent validation with different technologies, we identified a subset of miRNAs—most notably miR-638—with strong potential as endogenous normalizers. Importantly, our study not only identified miR-638 as a stable candidate but also demonstrated its practical applicability for normalization of independent fecal miRNA targets in a clinical cohort. Once confirmed in independent settings, these markers may enable the development of standardized protocols for fecal miRNA expression analysis and thereby support the reliable translation of miRNA biomarkers into clinical practice.

## Materials and methods

### Ethics

The study was conducted according to ethical principles for medical research involving human subjects as outlined in the World Medical Association Declaration of Helsinki. All human material samples, including blood and feces, were obtained with written informed consent. The study protocol was approved by the Institutional Review Board of Otto-von-Guericke University Magdeburg (number 99/10; 78/19 and 65/19).

### Ex vivo testing model

To analyze whether blood contaminates fecal samples, we developed a serial dilution model by mixing fecal samples with EDTA blood and serum. Each of the 6 dilution steps consists of 5 samples from IBD patients. The concentrations of blood and fecal samples are 100% fecal, 90% fecal/10% EDTA blood, 50% fecal/50% EDTA blood, 50% fecal/50% serum, 100% EDTA blood and 100% serum (Fig. 1A).

### Sample collection and cohorts

For the screening analysis we included fecal and blood specimen from 2 healthy subjects, which included 4 fecal (2 from each for biological validation) samples and 2 blood samples. To mimic the potential blood contamination the blood was added to the fecal samples in fixed proportion (*n* = 4). The total volume of the pure blood and fecal specimen was 100 µl per sample and the mixing proportions of the blood and stool samples are 20 µl of blood mixed with 100 µl of stool (Fig. 2A). The second cohort consists of stool samples from 30 patients with inflammatory bowel disease/ healthy controls: 10 patients with ulcerative colitis, 10 patients with Crohn’s disease and 10 healthy controls (IBP patient cohort, Fig. 3A). The third cohort consists of stool samples from liver patients with various diseases. Two subsets of this cohort were selected for this study: first, a cohort of stool samples from 59 patients with liver disease (LP cohort, Fig. 3A). 26 patients in this cohort had no evidence of liver cirrhosis, 4 patients had liver fibrosis, and 29 patients had liver cirrhosis. Of the 59 patients, 10 patients had alcohol associated liver disease (ALD), 39 patients had metabolic dysfunction associated liver disease (MASLD), and 2 patients had MASLD and increased alcohol intake (MetALD). The second subset of liver patients included 360 stool samples from 196 patients. 99 of the patients were included in the study at least twice, 52 patients were included at least three times, and in 14 patients could be included at 4 time points during the 2-year period. The distribution of liver disease in the second group of patients was structurally similar to that in the first group of patients. (Fig. 4A)

### MiRNA isolation

Extraction of total RNA and miRNA was performed with the Qiagen *miRNeasy* mini kit according to the manufacturer’s instructions (Qiagen, Hilden, Germany) as previously described by Lederer et al.^43^. The concentration and quality of miRNAs were determined photometrically.

### Microarray

For each cohort (10, 30 and 59 samples) one microarray experiment was performed, with the *Gene Chip microarray technology* by Affymetrix (Gene Chip Microarray 4.0, Affymetrix, Santa Clara, USA) according to the manufacturer’s instructions.

The analysis was performed according to Lederer et al. individually for each cohort (10, 30, 59 samples) using the *Transcriptome Analysis Console* (TAC).^44^ Only human miRNAs were included in the analyses. Quality control, background correction and normalization were conducted within TAC using the default workflow settings prior to downstream evaluation. The data were exported into csv for further statistical analysis.

### RT-PCR

Quantitative real-time PCR method was used in the serial dilution of blood in fecal samples and to verify the miRNA concentration in the stool samples. TaqMan miRNA assay technology.

(Thermo Fisher Scientific, Waltham, MA, USA) and SYBR Green method (Applied Biosystems, Life Technologies, Carlsbad, California, USA) were applied and 20ng of miRNA was measured using *BioRad CFX Cycler System* (BioRad, CA, USA) according to the manufacturer’s instructions. A spiked-in control with cel-miR 39 was used for normalization.^1,4,42^ The following primers were included: has-miR-638 (Assay-ID: 001582), cel-miR-39 (Assay-ID: 000200), hsa-miR-16-5p (Assay-ID: 000391), hsa-miR-21-5p (Assay-ID: 000397) (Thermo Fisher Scientific, Waltham, MA, USA).

### Statistical analysis

All statistical analyses were performed using *GraphPad Prism 9.0* (GraphPad, San Diego, CA, USA), and *R Studio* statistical software version 2022.12.0 (RStudio 2022.12.0 + 353 “Elsbeth Geranium”, Posit Software, PBC, Boston, USA). The Kruskal-Wallis test was used to assess significant differences between more than 2 non-parametric, statistically independent groups. The Benjamini-Hochberg method was used to determine the false discovery rate. The Spearman test was used to show correlations between potential normalizers and cofactors.

## Supplementary Information

Below is the link to the electronic supplementary material.

Supplementary Material 1

## Data Availability

All anonymized data will be included in the supplementary material upon acceptance. Patient related data will be available from the corresponding author at reasonable request.
